# Granger causality analysis reveals distinct spatio-temporal connectivity patterns in motor and perceptual visuo-spatial working memory

**DOI:** 10.3389/fncom.2014.00146

**Published:** 2014-11-13

**Authors:** Foteini Protopapa, Constantinos I. Siettos, Ioannis Evdokimidis, Nikolaos Smyrnis

**Affiliations:** ^1^School of Applied Mathematics and Physical Sciences, National Technical University of AthensAthens, Greece; ^2^Neurology Department, National and Kapodistrian University of Athens, Aeginition HospitalAthens, Greece; ^3^Laboratory of Sensorimotor Control, University Mental Health Research InstituteAthens, Greece; ^4^Psychiatry Department, National and Kapodistrian University of Athens, Aeginition HospitalAthens, Greece

**Keywords:** Granger causality, movement planning, change detection, EEG, functional connectivity

## Abstract

We employed spectral Granger causality analysis on a full set of 56 electroencephalographic recordings acquired during the execution of either a 2D movement pointing or a perceptual (yes/no) change detection task with memory and non-memory conditions. On the basis of network characteristics across frequency bands, we provide evidence for the full dissociation of the corresponding cognitive processes. Movement-memory trial types exhibited higher degree nodes during the first 2 s of the delay period, mainly at central, left frontal and right-parietal areas. Change detection-memory trial types resulted in a three-peak temporal pattern of the total degree with higher degree nodes emerging mainly at central, right frontal, and occipital areas. Functional connectivity networks resulting from non-memory trial types were characterized by more sparse structures for both tasks. The movement-memory trial types encompassed an apparent coarse flow from frontal to parietal areas while the opposite flow from occipital, parietal to central and frontal areas was evident for the change detection-memory trial types. The differences among tasks and conditions were more profound in α (8–12 Hz) and β (12–30 Hz) and less in γ (30–45 Hz) band. Our results favor the hypothesis which considers spatial working memory as a by-product of specific mental processes that engages common brain areas under different network organizations.

## Introduction

Understanding the mechanisms that pertain to the function of working memory (WM) is a fundamental and still open problem in neuroscience. WM is considered to have a key-role in higher-level cognitive processes such as decision making (Toth and Lewis, 1997), reasoning (Ruff et al., 2003), and recognition (Bledowski et al., 2012), since it is in charge of holding and simultaneously manipulating a small amount of information stored in the mind for a limited period of time for further processing. WM processing is considered to be governed by two functions, namely the phonological loop, and the visuo-spatial sketchpad (Baddeley and Hitch, 1974). Various studies have shown that the visuo-spatial sketchpad can be further separated depending on the task (“what” and “where” pathways) (Goodale et al., 1994). Furthermore, it has been shown that the mechanism of visuo-spatial WM retains complete motor programs that could either be executed or not (Awh and Jonides, 2001). It has been proposed that this dissociation for movement planning and visual processing for perception is present only when reaching and grasping of objects is performed in real time (Goodale and Westwood, 2004). Various studies that typically employ the S1–S2 paradigm have demonstrated the distinction of object vs. location information in spatial WM (see the review in Zimmer, 2008).

However, up to date, the question whether there are separate spatial WM processing streams in the brain for movement vs. spatial perception has not been resolved. In a recent study (Smyrnis et al., 2014) we tried to address this issue using scalp electroencephalographic recordings (EEG). The experiment involved memory and non-memory conditions on two tasks: one in which the spatial location of the target served as a goal for a 2D pointing movement (movement task) and one in which the spatial location of the target served as the onset of a perceptual yes/no decision (change detection task). We showed that there was a significant increase of the movement-related memory signal compared to the change detection-related memory signal in the central midline area in β and γ bands. Yet, the time-frequency analysis failed to identify in any region the opposite effect, namely an increase in the memory specific signal for the change detection task compared to the movement task, that would suggest a double dissociation for the two visuo-spatial WM processing streams (motor and perceptual). In a similar experiment (Srimal and Curtis, 2008), it has been compared the brain activity during a memory-guided saccade task between perceptual and motor spatial WM using fMRI. The authors reported no differences in the fronto-parietal network that was activated for both tasks.

In the current study, we make a step change and provide evidence that visuo-spatial information can be fully dissociated between different tasks (movement-planning vs. perceptual/change detection) and memory conditions (memory vs. non-memory). For that purpose, we calculated the functional directed connectivity networks between the recording sites via spectral Granger causality (GC) analysis. Spectral GC was employed to identify directions, strengths and frequencies of interactions between time-series on a set of 56 EEG electrodes.

## Materials and methods

### Experimental procedure

**Figure 1:**
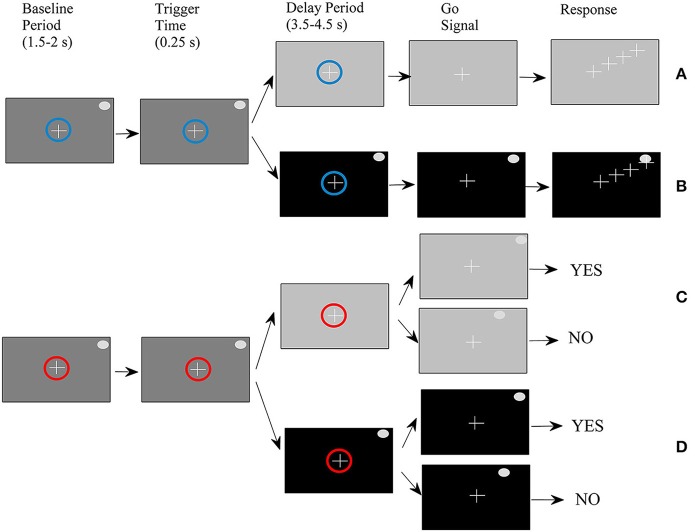
**Schematic of the experimental procedure**. **(A)** Movement-memory task (M-M), **(B)** Movement-non memory task (M-NM), **(C)** Change detection-memory task (CD-M), **(D)** Change detection-non memory task (CD-NM).

Four types of trials were performed by ten right-handed volunteers (seven men and three women) between the ages of 29 and 44 (mean age of 35) in a randomized order as described in a previous study (Smyrnis et al., 2014). All participants had no history of major medical or psychiatric illness and had normal or corrected-to-normal vision. They were fully informed about the experimental procedure and gave written informed consent. The study protocol was approved by the Aeginition Hospital Ethics committee and conformed to the 2013 WMA Declaration of Helsinki. For the sake of completeness of the presentation, we also describe it here briefly (see also Figure 1). At the beginning of each trial, a small circle was presented at the center of the monitor. The color of the circle was either blue indicating that the trial would be completed by making a pointing movement (movement task), or red indicating that the trial would be completed by pressing one of two buttons giving a yes/no response (change detection task). At the end of the baseline period which lasted randomly between 1500 and 2500 ms, a target was presented at one of 36 (equally distributed around 360° of an invisible circle) peripheral locations. After 250 ms (duration of target presentation) the background monitor color changed again either masking the peripheral target (memory condition) or allowing the target to remain visible (non-memory condition). After a delay period that varied randomly between 3500 and 4500 ms, the central circle disappeared; this served as a “go” signal for initiating a response.

**Figure 2:**
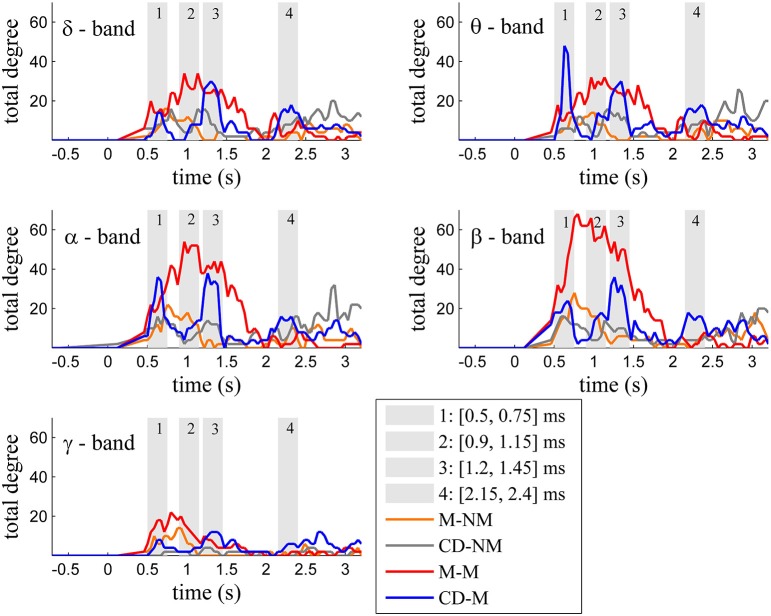
**Temporal evolution of the total degree of the binary networks whose connections represent statistically significant differences between the GC distributions of the delay and baseline period across frequency bands**. Four characteristic time intervals are marked with gray bars.

**Figure 3:**
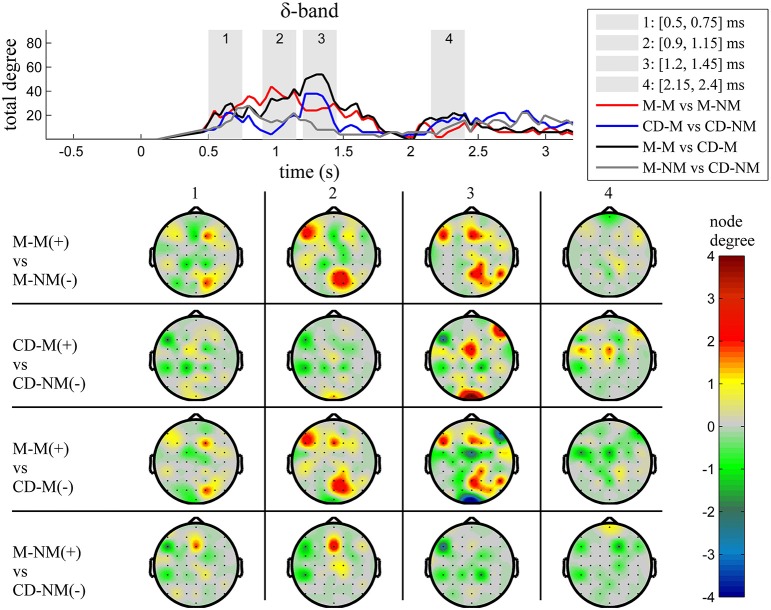
**δ band (1–4 Hz)**. **Top**: Temporal evolution of the total degree of the networks whose connections represent statistically significant differences in the GC distributions for each pair of electrodes, across four planned comparisons, namely, M-M vs. M-NM, CD-M vs. CD-NM, M-M vs. CD-M and M-NM vs. CD-NM trial types. Four characteristic time intervals are marked with gray bars (1: [0.5, 0.75] s, 2: [0.9, 1.15] s, 3: [1.25, 1.5] s, 4: [2, 2.25] s). **Bottom**: Topographic maps of node degrees of the statistically different networks resulted from the four planned comparisons, within each one of the characteristic time intervals.

**Figure 4:**
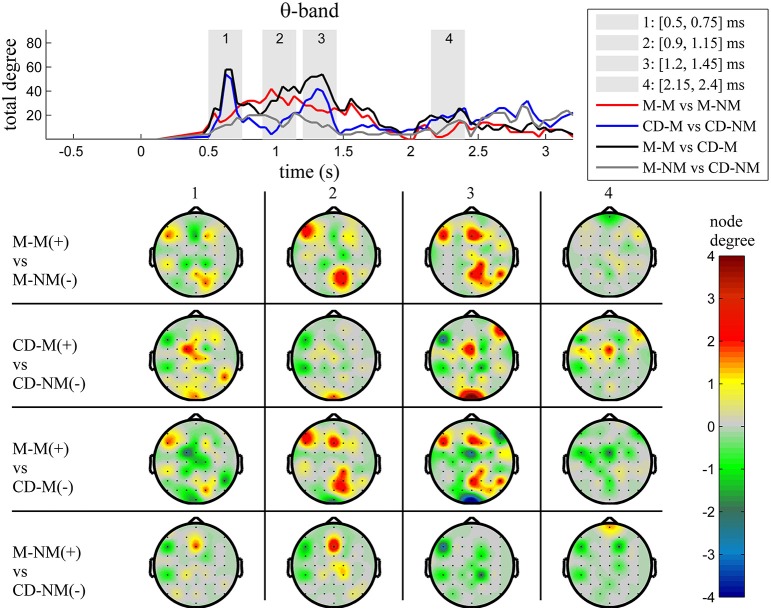
**θ band (4–8 Hz)**. **Top**: Temporal evolution of the total degree of the networks whose connections represent statistically significant differences in the GC distributions for each pair of electrodes, across four planned comparisons, namely, M-M vs. M-NM, CD-M vs. CD-NM, M-M vs. CD-M and M-NM vs. CD-NM trial types. Four characteristic time intervals are marked with gray bars (1: [0.5, 0.75] s, 2: [0.9, 1.15] s, 3: [1.25, 1.5] s, 4: [2, 2.25] s). **Bottom**: Topographic maps of node degrees of the statistically different networks resulted from the four planned comparisons, within each one of the characteristic time intervals.

For the movement task, the subjects had to simply place the cursor as fast as possible on the peripheral target disk by moving a joystick. For the change detection task, a second peripheral target appeared either at the same location as before (with 0.5 probability) or at another location and remained visible for 250 ms. Subjects had to press the “yes” vs. the “no” button depending on whether they perceived that the second target was at the same or different location as the first target. To summarize, we had four trial types: (1) movement-memory (M-M) (Figure 1A), (2) movement-non-memory (M-NM) (Figure 1B), (3) change detection-memory (CD-M) (Figure 1C), and (4) change detection-non memory (CD-NM) (Figure 1D).

For the recordings we used 56 scalp EEG electrodes. Horizontal and vertical eye movements were recorded using an infrared eye tracking system (IRIS SKALAR) attached to the head. The EEG and IRIS infrared analog signals were sampled at 1024 Hz using a data acquisition analog to digital card. The EEG signal recording for each trial began after the first 1000 ms of the baseline period and lasted until 1000 ms after the go signal and the beginning of the response period. During the recording session, the subject's head was immobilized with two side bars and a chin rest. The same A/D card sampled the X, Y signals that were produced from the analog joystick, as well as the TTL pulse signals of the joystick buttons used for the yes/no change detection response. The X, Y signal of the joystick was used to record the pointing movement trajectory from the central to the peripheral target. The time period from the “go” signal until the cursor was out of the central target was recorded as the RT for the movement trials. The time period from the “go” signal until the button press was recorded as the RT for the change detection trials. Each trial type was performed 72 times. The analysis of the EEG data was confined to 4.6 s (0.85 s baseline, 0.25 s target presentation and 3.5 s delay period).

### Data pre-processing

A total of 2880 trials were recorded, but we rejected 761 of them based on behavioral and electrophysiological criteria. As we described in a previous study (Smyrnis et al., 2014), the behavioral criteria that leaded to trial-rejection were: (1) reaction time below 100 ms or above 1500 ms and/or cursor exiting the center target at any time during baseline, target presentation and delay period; (2) directional error for the final endpoint of the movement (movement task) more than 30° clockwise or counter clockwise from the direction of the peripheral target and distance error larger than 60 pixels (1.4 inches) from the center of the peripheral target either overshooting or undershooting the peripheral target; (3) no button press after the “go” signal (change detection task). Using these criteria, 259 trials (9%, range for all subjects: 2.5–20%) were removed. The electrophysiological criteria that leaded to trial rejection were: (1) contamination of the EEG signal by the gross artifact (based on visual inspection of the signals); (2) trials in which the eyes during the baseline and delay periods did not remain fixed on the central target (based on the visual inspection of the electro-ocular signal recordings). Using these criteria, another 502 trials were removed (17.4%, range 0.5–31% for all subjects).

We also performed baseline-correction by subtracting from each EEG signal the mean of the first 250 ms of the baseline period. Overall, we ended up with 40 datasets (10 subjects × 4 trial types) of 56 multi time-series.

### Spectral GC analysis

Over the last years, GC analysis (Granger, 1969) has been applied in several studies, mainly in fMRI (see e.g., Roebroeck et al., 2005; Rypma et al., 2006; Sridharan et al., 2008; Florin et al., 2010; Gao et al., 2011; Hamilton et al., 2011; Liao et al., 2011; Miao et al., 2011; Zhou et al., 2011; Al-Aidroos et al., 2012; Tang et al., 2012; Wen et al., 2012; Wu et al., 2013; Zhang and Li, 2013; Sabatinelli et al., 2014; Yang and Shu, 2014) and (considerably fewer) in EEG (Chávez et al., 2003; Hesse et al., 2003; Gow et al., 2008; Keil et al., 2009; Dauwels et al., 2010; Barnett and Seth, 2011; Barrett et al., 2012; Nicolaou et al., 2012; de Tommaso et al., 2013; Wu et al., 2013).

Here, we employed the so-called spectral GC (Brovelli et al., 2004; Ding et al., 2006; Dauwels et al., 2010; Barrett et al., 2012) in sliding time-windows to construct the functional (causal) connectivity networks for all trial types in time-frequency domain. Spectral GC measures the proportion of power of a signal *x*, at a given frequency ω, that derives from its interaction with a signal *y* (Barrett et al., 2012) via: $F_{y\to x}(\omega )=\ln\left(1+\frac{H_{xy}(\omega )\sigma_{yy}{\text{H}}_{xy}^{*}(\omega )}{H_{xx}(\omega )\sigma_{xx}{\text{H}}_{xx}^{*}(\omega )}\right)$.

In the above equation, *H* is the inverse of the transfer matrix (i.e., the Fourier-transformed matrix of the model coefficients) and σ_xx_, σ_yy_ are the diagonal elements of the covariance matrix of residuals. More precisely, *H* is calculated via: $H(\omega )=inv[A(\omega )]=inv\left[I-\sum_{k\;=\;1}^{p}{\left(e^{-i2\pi \omega t_{0}}\right)}^{k}A_{k}\right]$, where *A_k_* are the model coefficients of the generalized Auto-Regressive model in time domain $(X(t)=\sum_{k\;=\;1}^{p}A_{k}X(t-kt_{0})+E_{x}(t))$, with *p* being the number of lags included in the Multivariate Regression model (Blinowska et al., 2004). Then, the covariance matrix of residuals is calculated via: $\Sigma \equiv \left(\begin{array}{cc} \sigma_{xx} & \sigma_{xy}\\ \sigma_{yx} & \sigma_{yy} \end{array}\right)=cov\left[\begin{array}{c} E_{x}(t)\\ E_{y}(t) \end{array}\right]$, where *E_x_*(*t*) and *E_y_*(*t*) are the residual errors of the AR model in time domain.

The number of lags *p* defines the Auto-Regressive model-order and is obtained using the Akaike Information Criterion (AIC) (Akaike, 1974) or the Bayesian Information Criterion (BIC) (Schwarz, 1978). Provided that BIC is considered to result to a better fit for neural applications compared to the AIC (Seth, 2010), we determined at each sliding-window the model order by minimizing the relation: $BIC(p)=2\log[\det(\tilde{\Sigma })]+\frac{2n^{2}p\log N_{total}}{N_{total}}$, where *n* is the number of variables, *N_total_* the total number of data points and $\tilde{\Sigma }$ the prediction error covariance matrix which is given by the equation $\tilde{\Sigma }=R(0)+\sum_{k\;=\;1}^{p}\hat{A}(k)R_{x}(k);\hat{A}(k)$; Â(*k*) contains the parameters of the MVAR model and can be estimated directly from the autocorrelation function of its output $R_{x}(k)=E[x(n)x^{T}(n+k)]$ by solving the multivariate Yule-Walker equations (Pereda et al., 2005; Krumin and Shoham, 2010): $\sum_{k\;=\;1}^{p}\hat{A}(j)R_{x}(i-k)=-R_{x}(i),1\;\leq \;i\;\leq \;p$.

At this point, we should note that according to the above formulation, *p* could be different at each sliding-window; here it was allowed to range between 1 and 20. The derived values of *p* ranged between 3 and 7 with a mean value of 4. All the values of the model order are in accordance with those found in previous studies and implementations of the method (e.g., Keil et al., 2009; Barnett and Seth, 2011; Nicolaou et al., 2012).

Finally, we should note that a necessary condition for the correct application of the method is the stationarity of the signals. The fact that we used a sliding-window approach facilitates the fulfillment of this requirement and also allows us to reveal any time-varying causal interactions. More precisely, we selected overlapping windows of 440 samples range (~430 ms) with a time sliding step corresponding to 44 samples (~43 ms). The selection of the time window was a compromise between the requirement for stationarity, time and frequency resolution. Thus, we ended up with 75 sliding-windows (8 in baseline, 1 in trigger presentation and 66 in delay period). Then, at every sliding-window we performed differencing (i.e., *x*(*t*)′ = *x*(*t*) − *x*(*t* − 1)) to ensure stationarity. The stationarity was tested by means of Kwiatkowski–Phillips–Schmidt–Shin test at a critical level of 0.01 (Kwiatkowski et al., 1992).

To test whether the derived GC pairwise values across frequencies were significant, we adopted a random permutation approach (Brovelli et al., 2004; Bollimunta et al., 2008). More specific, we constructed via time-replacement 100 surrogate data and calculated their GC values. We accepted as significant only the GC values that were higher from the 95% of the GC values of the surrogate data. Here, false discovery rate approximation was used in order to address the problem of multiple comparisons. All the above calculations were performed using Seth's GC Connectivity Analysis toolbox implemented in MATLAB (Seth, 2010).

Finally, due to the large quantity of datasets, the computational demands were significantly high and for that reason we used a 48 Quatro Core LINUX cluster with 3.2 GHz Intel Xeons and 4 GB RAM, for the parallelization of the procedure.

### Data analysis

The proposed analysis provided us, at each sliding-window, with a 56-dimensional matrix containing significant pairwise directed GC values, for each frequency in the range of [1, 45] Hz. The first step was to coarse-grain our data into frequency bands and thus obtain the so-called “band-limited GC” (Barrett et al., 2012) via: $F_{y\to x}(\omega_{1},\omega_{2})=\frac{1}{\omega_{2}-\omega_{1}}\int_{\omega_{1}}^{\omega_{2}}F_{y\to x}(\omega )d\omega ,\text{with }[\omega_{1},\omega_{2}]$, with [ω_1_, ω_2_ ] being the frequency range of each frequency band. Results were obtained at δ (1–4 Hz), θ (4–7 Hz), α (8–12 Hz), β (13–30 Hz) and γ (30–45 Hz) band. To sum up, (for every sliding-window, subject, trial type and frequency band) we derived the significant (non-zero) GC values between all nodes (channels). Having calculated these GC values we proceeded by testing whether the GC values in the delay period differ significantly from those in the baseline period. For this purpose we applied the non-parametric Wilcoxon test (with the significant threshold set at 0.02). The statistical test was performed at each sliding-window; to accommodate the problem of multiple comparisons in time we applied the so called cluster-based permutation algorithm (Maris and Oostenveld, 2007). Here, we used 200 permutations of the original data GC series. Firstly, we calculated the length of the sequential significant GC values of the original data. We then performed the non-parametric Wilcoxon test for each one of the 200 permutations, and rejected the sequential points of the original data as noise when their length was smaller than the 98% of the length distribution of the significant permuted sequential points.

For each one of the trial-types, we constructed the binary directed causal networks with edges obtained by the significant different from the baseline sequential GC values: for each specific task and condition, at each sliding window if the distribution (over subjects) of GC values between a pair of electrodes was determined to significantly differ from the one of the baseline then this connection was set to 1, zero otherwise. Here, at each sliding-window, we calculated the total degree of each resulted network.

We then proceeded with the implementation of a non-parametric Wilcoxon test at each sliding-window and frequency band to test whether there are significant differences in GC distributions (over subjects) in four planned comparisons between the trial types, namely, M-M vs. M-NM, CD-M vs. CD-NM, M-M vs. CD-M and M-NM vs. CD-NM. To adjust the problem of multiple comparisons, the resulting p-values were corrected using the false discovery rate (FDR) with *p* < 0.05 (Benjamini and Hochberg, 1995; Benjamini and Yekutieli, 2001; Groppe et al., 2011). This test resulted to eight binary directed networks whose connections represent statistically significant differences for the above mentioned planned comparisons.

For visualization purposes, the construction of those significant difference networks was as follows: assuming the comparison of two trial types A and B (e.g., M-M and CD-M), we tested for significant differences with respect to the distribution of GC values for each pair of electrodes, in both directions. For each direction, if the median of the distribution of GC values in A was significantly greater that the median of the distribution of GC in B then we assigned (as a convention) a (+1) flag to that directed connection; else if the median of the distribution of GC values in B was significantly greater that the median of the distribution of GC in A then we assigned a (−1) flag to that directed connection. In this manner we were able to extract the significant different “prevailed” networks: the network of A (B) trial type exhibiting statistically significant greater GC values (links) compared to the corresponding GC values of B (A) trial type. For visualization purposes, for each one of the four comparisons, we depict in the same topographic map the node degrees for each one of the significant different networks which were constructed with the above procedure.

## Results

Figure 2 depicts for each one of the five frequency bands (δ, θ, α, β, and γ) the evolution of the total degree of the GC networks for each trial type.

Within each trial-type, the temporal patterns of network total degrees for δ, θ, α, and β bands share common qualitative characteristics. In these frequency bands, the total network degree for the memory condition for both tasks (movement and change detection) was larger than the total degree of the corresponding non-memory condition. For the movement task, this difference was observed for a period of ~800 ms (in the interval [0.9–1.7] s) with a peak around 1 s (gray bar 2). For the change detection task, the total degree was bigger in the memory condition, over three smaller time intervals lasting ~250 ms each. These differences were profound early (gray bar 1), in the middle (gray bar 3) and late (gray bar 4) during the delay period (intervals [0.5, 0.75] s, [1.15, 1.4] s, [2.2, 2.45] s, respectively).

**Figure 5:**
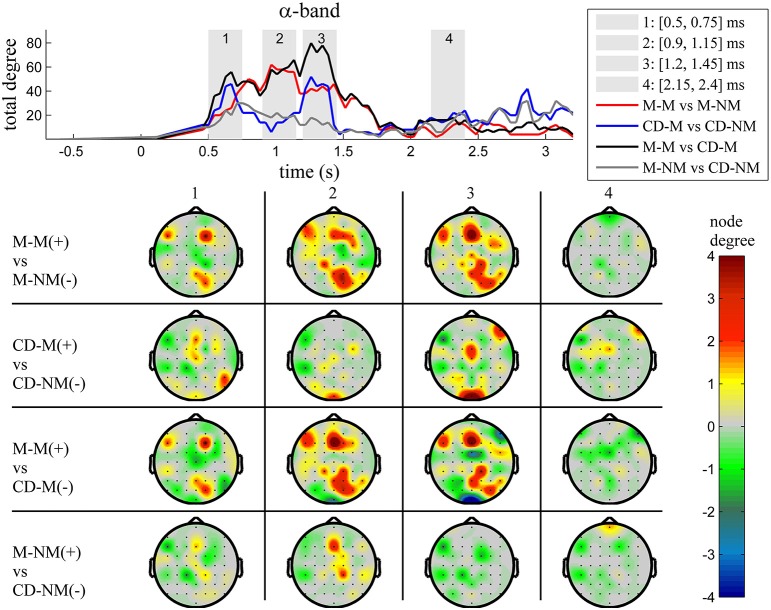
**α band (8–12 Hz)**. **Top**: Temporal evolution of the total degree of the networks whose connections represent statistically significant differences in the GC distributions for each pair of electrodes, across four planned comparisons, namely, M-M vs. M-NM, CD-M vs. CD-NM, M-M vs. CD-M and M-NM vs. CD-NM trial types. Four characteristic time intervals are marked with gray bars (1: [0.5, 0.75] s, 2: [0.9, 1.15] s, 3: [1.25, 1.5] s, 4: [2, 2.25] s). **Bottom**: Topographic maps of node degrees of the statistically different networks resulted from the four planned comparisons, within each one of the characteristic time intervals.

**Figure 6:**
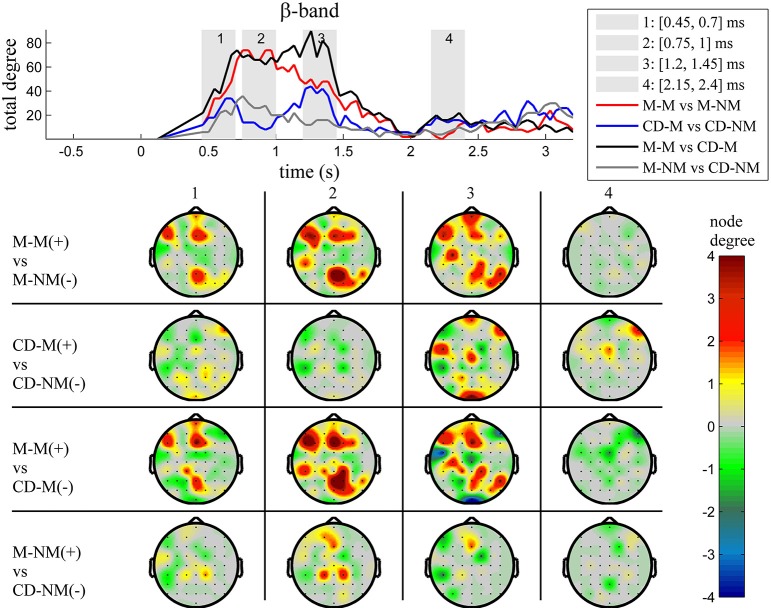
**β band (12–30 Hz)**. **Top**: Temporal evolution of the total degree of the networks whose connections represent statistically significant differences in the GC distributions for each pair of electrodes, across four planned comparisons, namely, M-M vs. M-NM, CD-M vs. CD-NM, M-M vs. CD-M and M-NM vs. CD-NM trial types. Four characteristic time intervals are marked with gray bars (1: [0.5, 0.75] s, 2: [0.9, 1.15] s, 3: [1.25, 1.5] s, 4: [2, 2.25] s). **Bottom**: Topographic maps of node degrees of the statistically different networks resulted from the four planned comparisons, within each one of the characteristic time intervals.

**Figure 7:**
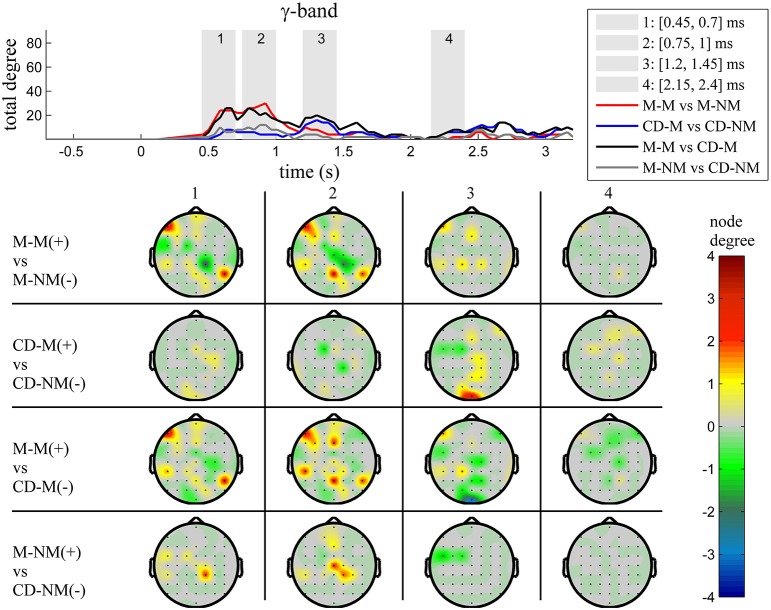
**γ band (30–45 Hz)**. **Top**: Temporal evolution of the total degree of the networks whose connections represent statistically significant differences in the GC distributions for each pair of electrodes, across four planned comparisons, namely, M-M vs. M-NM, CD-M vs. CD-NM, M-M vs. CD-M and M-NM vs. CD-NM trial types. Four characteristic time intervals are marked with gray bars (1: [0.5, 0.75] s, 2: [0.9, 1.15] s, 3: [1.25, 1.5] s, 4: [2, 2.25] s). **Bottom**: Topographic maps of node degrees of the statistically different networks resulted from the four planned comparisons, within each one of the characteristic time intervals.

The top Figures 3–**7** show the total degree for each binary network of significant differences between M-M and M-NM, CD-M and CD-NM, M-M and CD-M, M-NM and CD-NM across frequency bands. These results confirmed that the total degree of the causal connectivity networks over time during the delay period was significantly different across the four planned comparisons. As it is shown, significant differences were present at all frequency bands although they were more prominent at δ, θ, α, and β and less so at γ band.

We also depict topographic maps, for four characteristic time periods as marked with gray bars in the corresponding top figure, of the node degrees for each one of the significant different networks which were constructed with the procedure described in the methods section. These maps depict the spatial distribution of the average significant differences, over the corresponding time periods and were created with the aid of FieldTrip (Oostenveld et al., 2011). The M-M trials exhibited significantly higher degree nodes at the central and left frontal electrodes as well as right-parietal electrodes compared to the M-NM trials. The CD-M trials exhibited higher degree nodes at central, right frontal and occipital electrodes compared to the CD-NM trials. The significant difference in the spatial topography of the memory networks for both tasks is revealed in the comparison between the M-M and CD-M trial types for all frequency bands. It should be noted here that these different (mostly non-overlapping) topographic patterns were related specifically to the memory conditions since the corresponding comparison between the M-NM and CD-NM trial types did not reveal analogous pattern formations.

Quantitatively, compared to the lower frequency bands (δ and θ, Figures 3, 4), the differences in the networks were larger in α (Figure 5) and profoundly in β (Figure 6) band. At γ band, no profound differences were observed (Figure 7).

**Figure 8:**
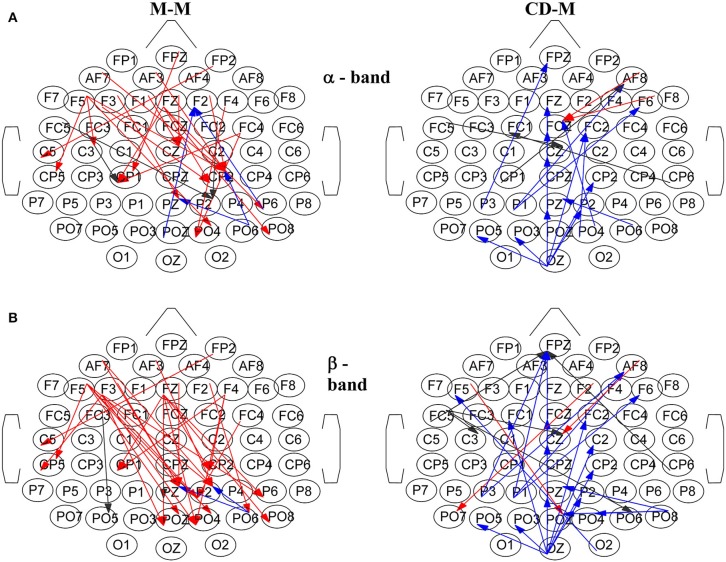
**Characteristic snapshots of the directed functional causal connectivity networks for the movement-memory (M-M) and change detection-memory (CD-M) trial types for: **(A)** α band at ~1 s for the M-M and at ~1.3 s for the CD-M, **(B)** β band at ~0.9 s for the M-M and at ~1.3 s for the CD-M**. Left column corresponds to M-M and right column to CD-M. The directed connections are colored with red, black and blue depending from which area are originated (frontal, central and parietal, respectively).

Figure 8 illustrates characteristic snapshots of the resulting directed functional networks for the M-M and CD-M trial types taken at the maxima of the corresponding total degrees of α band (Figure 8A) and β band (Figure 8B). As it is shown, the connectivity of the M-M differs vastly from that of CD-M (actually they are mostly non-overlapping). In the case of M-M there is an apparent coarse flow from the frontal to the parietal nodes. On the other hand, the functional connectivity of the CD-M is characterized by a coarse flow from occipital and parietal to central and frontal areas.

## Discussion

### Comparison with previous studies

This study addressed the question whether there are separate spatial WM processing streams in the brain for movement vs. spatial perception using two memory tasks. In our previous study using the same data set (Smyrnis et al., 2014) we examined the behavioral performance of these two memory tasks and showed that the memorization of the target location in the movement and the change detection tasks did not have any different effect on reaction time or loss of spatial accuracy, thus favoring the hypothesis of a common mechanism of spatial WM as these tasks are concerned. Amplitude spectrum analysis of the EEG, in that same study (Smyrnis et al., 2014) revealed that the α (8–12 Hz) band signal was smaller while the β (13–30 Hz) and γ (30–45 Hz) band signals were larger in the memory compared to the non-memory condition. The α band signal difference was confined to the frontal midline area; the β band signal difference extended over the right hemisphere and midline central area and the γ band signal difference was confined to the right occipito-parietal area. Importantly, both in β and γ bands, we observed a significant increase of the movement-related compared to the perceptual-related memory specific amplitude spectrum signal in the central midline area. The amplitude spectrum analysis of the EEG signal though failed to detect the opposite effect, namely an increase in the memory specific signal for the change detection task compared to the movement task that would provide evidence for a double dissociation.

The same question of separate processing streams for perception and movement in spatial WM has been addressed before (Srimal and Curtis, 2008), but with two important differences: firstly the movement to be performed at the memorized spatial location was a saccade and not an arm pointing movement and secondly, the measurement of the BOLD signal of fMRI instead of the EEG signal. Again, the analysis did not show differences in the amplitude of the BOLD signal that was similarly increased for the change detection and the saccadic movement task during the delay period in a fronto-parietal network.

The major difference of the two previous studies mentioned above and the present study is that these studies measured the amplitude of the brain signals during the memory delay period in the two tasks of spatial WM, whether that being EEG signals (Smyrnis et al., 2014) or hemodynamic response signals (BOLD) (Srimal and Curtis, 2008). In this study, instead of looking at the amplitude signals *per se*, we measured the connectivity patterns of these signals using spectral GC. Using this method, a double dissociation of spatial WM for movements and perception was revealed following the dissociation of vision for perception and vision for movement that was described in the introduction. This clear dissociation emerged at the level of functional connectivity both in time and in space.

### Functional connectivity network for the memory movement task

Memorizing a target location for the planning and execution of a pointing movement resulted in the emergence of a robust pattern characterized by relatively higher (compared to the non-memory condition) total network degree extending over the first 2 s of the delay period. The pattern of significant node degrees occupied mainly the frontal and right-parietal areas. This network modulation was evident for all frequency bands but was most evident in β band and least evident in γ band. The functional connectivity networks of the M-M trial types revealed an apparent coarse flow from frontal to parietal area suggesting that the parietal area might be essential to the continuous updating of the memorized motor plan for the upcoming movement. In line with this hypothesis we have shown in a previous study (Smyrnis et al., 2003) that transcranial brain stimulation of the parietal but not prefrontal cortex in healthy human volunteers early during the memory delay period resulted in the disruption of the memorized movement plan (lower accuracy of the upcoming movement).

### Functional connectivity network for the change detection task

Memorizing a target location for the performance of a change detection task also resulted in the emergence of a robust pattern characterized by higher network degrees compared to the non-memory condition. Here, the increase in the total network degree followed a very different temporal evolution compared to that observed for the memorization process in the pointing movement task. Three distinct peaks were observed occurring early, in the middle and late during the delay period. The topography of the node degrees for the CD-M was also clearly different from that observed for the M-M. Higher degree nodes were now observed mainly at occipital, central and right frontal areas. The particular network modulation was again evident for all frequency bands but was most evident in β band and least evident in γ band.

Functional connectivity networks for the CD-M trial type revealed a very different network organization exhibiting a coarse flow from occipital and parietal to central and frontal areas. In this task, the location of the target had to be maintained in WM to serve for a decision process after the delay period. This decision is based on an abstract stimulus response mapping rule where the subject has to respond yes (by pressing one button) if the S2 target location matches the S1 target location, or no (by pressing another button) if S2 does not match S1. Then this is a delayed matching to sample task where the prefrontal cortex is crucial for maintaining both the stimulus location information and the information about the rule for responding (Fuster, 1997; Owen, 1997; Petrides, 2005; Zimmer, 2008; Michels et al., 2010). A flow of information from occipital and parietal to the frontal areas might serve the continuous updating of this information in the frontal areas during the delay period.

### Network hypothesis for spatial WM

We believe that our results favor the hypothesis of the brain function as a network of neural structures that organizes depending on the task-specific demand (Postle, 2006; Zimmer, 2008). In this view, there is no specific subsystem dedicated to spatial WM. Instead, we could speak of different tasks (change detection or pointing movement) with specific demands. Each one of these tasks is associated with mental operations running on representations. These operations are realized by specific neural networked structures that are domain-specific (Zimmer, 2008).

An important issue that deserves further discussion is that the study of WM functions is fruitful if one views these mental operations as being the result of activation of specific spatio-temporal brain networks (Zimmer, 2008). This view is within the general frame of looking at the brain as a connectome, a frame that is becoming increasingly popular in functional neuroimaging (Hesse et al., 2003; Bressler et al., 2008; Friston, 2009; Amini et al., 2010; Havlicek et al., 2010; Joyce et al., 2010; Rubinov and Sporns, 2010; Bressler and Seth, 2011; Jiao et al., 2011; Krueger et al., 2011; Zuo et al., 2011; Ge et al., 2012). The use of GC as a method to reveal the spatio-temporal functional connectivity patterns in EEG signals during the performance of cognitive tasks seems to be very promising in this domain. Over the last few years there is an increasing interest in studying the dynamics of brain cognitive processes using GC methodologies [see for example in EEG data, (Gow et al., 2008; Keil et al., 2009)].

## Conclusion

This study showed that the spatial WM for locations can be dissociated to distinct spatio-temporal functional causal connectivity networks of EEG activity depending on whether the memorized spatial location will be used for a perceptual change detection task or whether the spatial location will be the target for a pointing arm movement. Thus, the successful use of GC for the identification of these distinct network patterns that could potentially serve as “functional biomarkers” suggests that this method could be a powerful tool to study complex cognitive functions using scalp recorded EEG in both healthy subjects and patients with neuropsychiatric disorders.

### Conflict of interest statement

The authors declare that the research was conducted in the absence of any commercial or financial relationships that could be construed as a potential conflict of interest.

## Abbreviations

AIC: Akaike Information Criterion
BIC: Bayesian Information Criterion
CD-M: Change detection-memory
CD-NM: Change detection-non memory
EEG: Electroencephalography
fMRI: functional Magnetic Resonance Imaging
GC: Granger causality
M-M: Movement-memory
M-NM: Movement-non memory
WM: Working memory.

## References

[B1] Akaike H. (1974). A new look at the statistical model identification. IEEE Trans. Automat. Control 19, 716–723 10.1109/TAC.1974.1100705

[B2] Al-Aidroos N. Said C. P. Turk-Browne N. B. (2012). Top-down attention switches coupling between low-level and high-level areas of human visual cortex. Proc. Natl. Acad. Sci. U.S.A. 109, 14675–14680. 10.1073/pnas.1202095109 22908274 PMC3437858

[B3] Amini L. Jutten C. Achard S. David O. Kahane P. Vercueil L.. (2010). Comparison of five directed graph measures for identification of leading interictal epileptic regions. Physiol. Meas. 31, 1529–1546. 10.1088/0967-3334/31/11/009 20952817 PMC3368828

[B4] Awh E. Jonides J. (2001). Overlapping mechanisms of attention and spatial working memory. Trends Cogn. Sci. 5, 119–126. 10.1016/S1364-6613(00)01593-X 11239812

[B5] Baddeley A. D. Hitch G. J. (1974). Working memory, in Recent Advances in Learning and Motivation, ed Bower G. A. (New York, NY: Academic Press), 47–89.

[B6] Barnett L. Seth A. K. (2011). Behaviour of Granger causality under filtering: theoretical invariance and practical application. J. Neurosci. Methods 201, 404–419. 10.1016/j.jneumeth.2011.08.010 21864571

[B7] Barrett A. B. Murphy M. Bruno M.-A. Noirhomme Q. Boly M. Laureys S.. (2012). Granger causality analysis of steady-state electroencephalographic signals during propofol-induced anaesthesia. PLoS ONE 7:e29072. 10.1371/journal.pone.0029072 22242156 PMC3252303

[B8] Benjamini Y. Hochberg Y. (1995). Controlling the false discovery rate: a practical and powerful approach to multiple testing. J. R. Stat. Soc. Ser. B (Methodol.) 57, 289–300.

[B9] Benjamini Y. Yekutieli D. (2001). The control of the false discovery rate in multiple testing under dependency. Ann. Stat. 29, 1165–1188. 10.1214/aos/101369999818298808

[B10] Bledowski C. Kaiser J. Wibral M. Yildiz-Erzberger K. Rahm B. (2012). Separable neural bases for subprocesses of recognition in working memory. Cereb. Cortex 22, 1950–1958. 10.1093/cercor/bhr276 21965439

[B11] Blinowska K. Kuś R. Kamiński M. (2004). Granger causality and information flow in multivariate processes. Phys. Rev. E 70:050902. 10.1103/PhysRevE.70.050902 15600583

[B12] Bollimunta A. Chen Y. Schroeder C. E. Ding M. (2008). Neuronal mechanisms of cortical alpha oscillations in awake-behaving macaques. J. Neurosci. 28, 9976–9988. 10.1523/JNEUROSCI.2699-08.2008 18829955 PMC2692971

[B13] Bressler S. L. Seth A. K. (2011). Wiener-Granger causality: a well established methodology. Neuroimage 58, 323–329. 10.1016/j.neuroimage.2010.02.059 20202481

[B14] Bressler S. L. Tang W. Sylvester C. M. Shulman G. L. Corbetta M. (2008). Top-down control of human visual cortex by frontal and parietal cortex in anticipatory visual spatial attention. J. Neurosci. 28, 10056–10061. 10.1523/JNEUROSCI.1776-08.2008 18829963 PMC2583122

[B15] Brovelli A. Ding M. Ledberg A. Chen Y. Nakamura R. Bressler S. L. (2004). Beta oscillations in a large-scale sensorimotor cortical network: directional influences revealed by Granger causality. Proc. Natl. Acad. Sci. U.S.A. 101, 9849–9854. 10.1073/pnas.0308538101 15210971 PMC470781

[B16] Chávez M. Martinerie J. Le Van Quyen M. (2003). Statistical assessment of nonlinear causality: application to epileptic EEG signals. J. Neurosci. Methods 124, 113–128. 10.1016/S0165-0270(02)00367-9 12706841

[B17] Dauwels J. Vialatte F. Musha T. Cichocki A. (2010). A comparative study of synchrony measures for the early diagnosis of Alzheimer's disease based on EEG. Neuroimage 49, 668–693. 10.1016/j.neuroimage.2009.06.056 19573607

[B18] de Tommaso M. Stramaglia S. Marinazzo D. Trotta G. Pellicoro M. (2013). Functional and effective connectivity in EEG alpha and beta bands during intermittent flash stimulation in migraine with and without aura. Cephalalgia 33, 938–947. 10.1177/0333102413477741 23439574

[B19] Ding M. Chen Y. Bressler S. L. (2006). Granger causality: basic theory and application to neuroscience, in Handbook of Time Series Analysis, eds Schelter B. Winterhalder M. Timmer J. (Berlin: Wiley-VCH Verlage), 451–474.

[B20] Florin E. Gross J. Pfeifer J. Fink G. R. Timmermann L. (2010). The effect of filtering on Granger causality based multivariate causality measures. Neuroimage 50, 577–588. 10.1016/j.neuroimage.2009.12.050 20026279

[B21] Friston K. (2009). Causal modelling and brain connectivity in functional magnetic resonance imaging. PLoS Biol. 7:e33. 10.1371/journal.pbio.1000033 19226186 PMC2642881

[B22] Fuster J. M. (1997). Network memory. Trends Neurosci. 20, 451–459 10.1016/S0166-2236(97)01128-4 9347612

[B23] Gao Q. Duan X. Chen H. (2011). Evaluation of effective connectivity of motor areas during motor imagery and execution using conditional Granger causality. Neuroimage 54, 1280–1288. 10.1016/j.neuroimage.2010.08.071 20828626

[B24] Ge T. Cui Y. Lin W. Kurths J. Liu C. (2012). Characterizing time series: when Granger causality triggers complex networks. N. J. Phys. 14:083028 10.1088/1367-2630/14/8/083028

[B25] Goodale M. A. Meenan J. P. Bulthoff H. H. Nicolle D. A. Murphy K. J. Racicot C. I. (1994). Separate neural pathways for the visual analysis of object shape in perception and prehension. Curr. Biol. 14, 604–610. 10.1016/S0960-9822(00)00132-9 7953534

[B26] Goodale M. A. Westwood D. A. (2004). An evolving view of duplex vision: separate but interacting cortical pathways for perception and action. Curr. Opin. Neurobiol. 14, 203–211. 10.1016/j.conb.2004.03.002 15082326

[B27] Gow D. W. Jr. Segawa J. A. Ahlfors S. P. Lin F.-H. (2008). Lexical influences on speech perception: a Granger causality analysis of MEG and EEG sources estimates. Neuroimage 43, 614–623. 10.1016/j.neuroimage.2008.07.027 18703146 PMC2585985

[B28] Granger C. (1969). Investigating causal relations by econometric models and cross-spectral methods. Econometrica 37, 424–438 10.2307/1912791

[B29] Groppe D. M. Urbach T. P. Kutas M. (2011). Mass univariate analysis of event-related brain potentials/fields I: a critical tutorial review. Psychophysiology 48, 1711–1725. 10.1111/j.1469-8986.2011.01273.x 21895683 PMC4060794

[B30] Hamilton P. J. Chen G. Thomason M. E. Schwartz M. E. Gotlib I. H. (2011). Investigating neural primacy in major depressive disorder: multivariate Granger causality analysis of resting-state fMRI time-series data. Mol. Psychiatry 16, 763–772. 10.1038/mp.2010.46 20479758 PMC2925061

[B31] Havlicek M. Jan J. Brazdil M. Calhoun V. D. (2010). Dynamic Granger causality based on Kalman filter for evaluation of functional network connectivity in fMRI data. Neuroimage 53, 65–77. 10.1016/j.neuroimage.2010.05.063 20561919 PMC4347842

[B32] Hesse W. Möller E. Arnold M. Schack B. (2003). The use of time-variant EEG Granger causality for inspecting directed interdependencies of neural assemblies. J. Neurosci. Methods 124, 27–44. 10.1016/S0165-0270(02)00366-7 12648763

[B33] Jiao Q. Lu G. Zhang Z. Zhong Y. Wang Z. Guo Y.. (2011). Granger causal influence predicts BOLD activity levels in the default mode network. Hum. Brain Mapp. 32, 154–161. 10.1002/hbm.21065 21157880 PMC6870036

[B34] Joyce K. E. Laurienti P. J. Burdette J. H. Hayasaka S. (2010). A new measure of centrality for brain networks. PLoS ONE 5:e12200. 10.1371/journal.pone.0012200 20808943 PMC2922375

[B35] Keil A. Sabatinelli D. Ding M. Lang P. J. Ihssen N. Heim S. (2009). Re-entrant projections modulate visual cortex in affective perception: evidence from Granger causality analysis. Hum. Brain Mapp. 30, 532–540. 10.1002/hbm.20521 18095279 PMC3622724

[B36] Krueger F. Landgraf S. van der Meer E. Deshpande G. Hu X. (2011). Effective connectivity of the multiplication network: a functional MRI and multivariate Granger causality mapping study. Hum. Brain Mapp. 32, 1419–1431. 10.1002/hbm.21119 20715080 PMC6870371

[B37] Krumin M. Shoham S. (2010). Multivariate autoregressive modeling and granger causality analysis of multiple spike trains. Comput. Intell. Neurosci. 2010:752428. 10.1155/2010/752428 20454705 PMC2862319

[B38] Kwiatkowski D. Phillips P. C. B. Schmidt P. Shin Y. (1992). Testing the null hypothesis of stationarity against the alternative of a unit root. J. Econom. 54, 159–178 10.1016/0304-4076(92)90104-Y

[B39] Liao W. Ding J. Marinazzo D. Xu Q. Wang Z. Yuan C.. (2011). Small-world directed networks in the human brain: multivariate Granger causality analysis of resting-state fMRI. Neuroimage 54, 2683–2694. 10.1016/j.neuroimage.2010.11.007 21073960

[B40] Maris E. Oostenveld R. (2007). Nonparametric statistical testing of EEG- and MEG-data. J. Neurosci. Methods 164, 177–190. 10.1016/j.jneumeth.2007.03.024 17517438

[B41] Miao X. Wu X. Li R. Chen K. Yao L. (2011). Altered connectivity pattern of hubs in default-mode network with Alzheimer's disease: a Granger causality modeling approach. PLoS ONE 6:e25546. 10.1371/journal.pone.0025546 22022410 PMC3191142

[B42] Michels L. Bucher K. Luchinger R. Klaver P. Martin E. Jeanmonod D.. (2010). Simultaneous EEG-fMRI during a working memory task: modulations in low and high frequency bands. PLoS ONE 5:e10298. 10.1371/journal.pone.0010298 20421978 PMC2858659

[B43] Nicolaou N. Hourris S. Alexandrou P. Georgiou J. (2012). EEG-based automatic classification of “awake” versus “anesthetized” state in general anesthesia using Granger causality. PLoS ONE 7:e33869. 10.1371/journal.pone.0033869 22457797 PMC3310868

[B44] Oostenveld R. Fries P. Maris E. Schoffelen J.-M. (2011). FieldTrip: open source software for advanced analysis of MEG, EEG, and invasive electrophysiological data. Comput. Intell. Neurosci. 2011:156869. 10.1155/2011/156869 21253357 PMC3021840

[B45] Owen A. M. (1997). The functional organization of working memory processes within lateral frontal cortex: the contribution of functional neuroimaging. Eur. J. Neurosci. 9, 1329–1339. 10.1111/j.1460-9568.1997.tb01487.x 9240390

[B46] Pereda E. Quiroga R. Q. Bhattacharya J. (2005). Nonlinear multivariate analysis of neurophysiological signals. Prog. Neurobiol. 77, 1–37. 10.1016/j.pneurobio.2005.10.003 16289760

[B47] Petrides M. (2005). Lateral prefrontal cortex: architectonic and functional organization. Philos. Trans. R. Soc. B Biol. Sci. 360, 781–795. 10.1098/rstb.2005.1631 15937012 PMC1569489

[B48] Postle B. R. (2006). Working memory as an emergent property of the mind and brain. Neuroscience 139, 23–38. 10.1016/j.neuroscience.2005.06.005 16324795 PMC1428794

[B49] Roebroeck A. Formisano E. Goebel R. (2005). Mapping directed influence over the brain using Granger causality and fMRI. Neuroimage 25, 230–242. 10.1016/j.neuroimage.2004.11.017 15734358

[B50] Rubinov M. Sporns O. (2010). Complex network measures of brain connectivity: uses and interpretations. Neuroimage 52, 1059–1069. 10.1016/j.neuroimage.2009.10.003 19819337

[B51] Ruff C. C. Knauff M. Fangmeier T. Spreer J. (2003). Reasoning and working memory: common and distinct neuronal processes. Neuropsychologia 41, 1241–1253. 10.1016/S0028-3932(03)00016-2 12753963

[B52] Rypma B. Berger J. S. Prabhakaran V. Bly B. M. Kimberg D. Y. Biswal B. B.. (2006). Neural correlates of cognitive efficiency. Neuroimage 33, 969–979. 10.1016/j.neuroimage.2006.05.065 17010646

[B53] Sabatinelli D. McTeaque L. M. Dhamala M. Frank D. W. Wanger T. J. Adhikati B. M. (2014). Reduced medial prefrontal-subcortical connectivity in dysphoria: Granger causality analyses of rapid functional MRI. Brain Connect. [Epub ahead of print]. 10.1089/brain.2013.0186 24575774

[B54] Schwarz G. (1978). Estimating the dimension of a model. Ann. Stat. 6, 461–464 10.1214/aos/1176344136

[B55] Seth A. K. (2010). A MATLAB toolbox for Granger causal connectivity analysis. J. Neurosci. Methods 186, 262–273. 10.1016/j.jneumeth.2009.11.020 19961876

[B56] Smyrnis N. Protopapa F. Tsoukas E. Balogh A. Siettos C. I. Evdokimidis I. (2014). Amplitude spectrum EEG signal evidence for the dissociation of motor and perceptual spatial working memory in the human brain. Exp. Brain Res. 232, 659–673. 10.1007/s00221-013-3774-z 24281356

[B57] Smyrnis N. Theleritis C. Evdokimidis I. Müri R. M. Karandreas N. (2003). Single-pulse transcranial magnetic stimulation of parietal and prefrontal areas in a memory delay arm pointing task. J. Neurophysiol. 89, 3344–3350. 10.1152/jn.00810.2002 12783961

[B58] Sridharan D. Levitin D. J. Menon V. (2008). A critical role for the right fronto-insular cortex in switching between central-executive and default-mode networks. Proc. Natl. Acad. Sci. U.S.A. 105, 12569–12574. 10.1073/pnas.0800005105 18723676 PMC2527952

[B59] Srimal R. Curtis C. E. (2008). Persistent neural activity during maintenence of spatial position in working memory. Neuroimage 39, 455–468. 10.1016/j.neuroimage.2007.08.040 17920934 PMC2219966

[B60] Tang W. Bressler S. L. Sylvester C. M. Shulman G. L. Corbetta M. (2012). Measuring Granger causality between cortical regions from voxelwise fMRI BOLD signals with LASSO. PLoS Comput. Biol. 8:e1002513. 10.1371/journal.pcbi.1002513 22654651 PMC3359965

[B61] Toth J. Lewis C. (1997). The role of working memory and external representation in individual decision making, in AAAI Technical Report (Menlo Park, CA), 109–115.

[B62] Wen X. Yao L. Liu Y. Ding M. (2012). Causal interactions in attention networks predict behavioral performance. J. Neurosci. 32, 1284–1292. 10.1523/JNEUROSCI.2817-11.2012 22279213 PMC6796284

[B63] Wu G.-R. Liao W. Stramaglia S. Chen H. Marinazzo D. (2013). Recovering directed networks in neuroimaging datasets using partially conditioned Granger causality. Brain Connect. 3, 294–301. 10.1089/brain.2013.0142 23530810 PMC3685317

[B64] Yang J. Shu H. (2014). The causal interactions between bilateral M1 and SMA during verb comprehension, motor imagery and hand motion. Arch. Neurosci. 2:e18185 10.5812/archneurosci.18185

[B65] Zhang H. Li X. (2013). Effective connectivity of facial expression network by using Granger causality analysis, in Parallel Processing of Images and Optimization and Medical Imaging Processing, Vol. 8920, ed Liuo J. (Wuhan), 89200K.

[B66] Zhou Z. Wang X. Klahr N. J. Liu W. Arias D. Liu H.. (2011). A conditional Granger causality model approach for group analysis in functional magnetic resonance imaging. Magn. Reson. Imaging 29, 418–433. 10.1016/j.mri.2010.10.008 21232892 PMC3063394

[B67] Zimmer H. D. (2008). Visual and spatial working memory: from boxes to networks. Neurosci. Biobehav. Rev. 32, 1373–1395. 10.1016/j.neubiorev.2008.05.016 18603299

[B68] Zuo X.-N. Ehmke R. Mennes M. Imperati D. Castellanos F. X. Sporns O.. (2011). Network centrality in the human functional connectome. Cereb. Cortex 22, 1862–1875. 10.1093/cercor/bhr269 21968567

